# Indents

**Published:** 1905-09-15

**Authors:** 


### INDENTS.

☞ **Brief Articles of Technical or Scientific Value will receive notice and comment. Contributions invited.**

**Poison Ivy.**

℞ Acetate of lead.........................grs x
Spirit of nitrous ether......................℥j
Sig. Apply to affected parts as a lotion.—
W. L. Hutchinson, M. D. in *Medical Brief*.

**Pumice Holders.** There is nothing better to hold pumice and peroxide of hydrogen, mixed to clean teeth with, than a round watch glass with thick sides at right angles to the bottom and about two and one-half inches in diameter.

**Burns from Acids.** If you get carbolic acid on any surface where it is not wanted, apply absolute alcohol at once. If hydrofluoric acid, apply a strong solution of bicarbonate of soda. Never use these acids without having readily at hand some agent to stop their action immediately in case of accident.—*Review*.

**Easy Method of Removing Regulating Bands.** To remove regulating bands or crowns, grasp the band with forceps and squeeze with firm pressure, repeating the process around the tooth if possible. This will loosen the cement and may even expand the band so that it can be easily removed.—F. W. Stephan, *Review*.

**Treatment for Pyorrhea.** Cauterize the diseased sockets with the thermo-cautery, and then inject between the root and alveolus a solution of bichloride of proper strength in distilled water. This treatment supplemented by removal of deposits and firmly splinting the teeth to prevent mobility, is earnestly recommended by M. Barrie, of Paris.

**Copper Sulphate for Pyorrhea.** After scaling the teeth, pack crystal or copper sulphate in the pockets by means of a pointed stick in a porte polisher. Dip the point in oil of cassia and while moist touch it to some fine crystals of the copper sulphate and carry them to the bottom of the pockets. In bad cases make an application twice each week.—*Review.*

**Tempering Instruments.**

| Temperatures. | Color. | Use. |
|---|---|---|
| 220 C. 428 F. | Pale yellow | Surgical Instruments. |
| 230 C. 446 F | Straw | Knives, razors, wood tools. |
| 255 C. 491 F | Brown yellow | Chisels and scissors. |
| 265 C. 509 F | Purplish | Axes, heavy knives. |
| 275 C. 527 F | Purple | Table knives and springs. |
| 290 C. 534 F | Pale blue | Watch springs, swords. |
| 295 C. 563 F | Dark blue | Fine saws and drills. |
| 315 C. 600 F | Very dark blue | Hand saws. |
| 350 C. 662 F | Very dark blue-green | Too soft for tools. |

**To Make a Nice Pink Gum.** If you want the pink gum to be an equal distance from the necks of teeth so as to show a well-defined line instead of an irregular or hit or miss line between the pink rubber gum and rubber above it that is used in the plate, invest the waxed-up model, in part of flask that hold model, only as high as you wish the pink gum wide. Pack pink rubber after separating as high as your investment in front. If you have invested so as to get a well-defined line, your pink gum will show a defined line also.—*Summary.*

**Wearing Out or Rusting Out.** An old adage says, "It is better to wear out than to rust out," but a good many who fully appreciate the truth of this are really "rusting out," without knowing it. They are in a rut where they plod along day after day, following the same rules of practice and using the same class of appliances with which they were equipped when first licensed to practice. The "rust" is corroding the metal of their ability deeper and deeper every day, and fondly believing they are "wearing out"

in an honest endeavor to do the best they can, they make no effort at self-analysis and never attain an appreciation of the difference between "rust" and "wear."—*Pacific Gazette.*

**Pyorrhea Remedies.** Dr. Black says in the application of remedies for the treatment of pyorrhea, no caustic remedies should be employed, except, possibly, when disinfection is to be accomplished. Stimulating antiseptics are always admirable and serve to stimulate cell-development and create repair tissue, while keeping the wound free from bacterial interference. The mouth is not so liable to infection as other organs probably because the tissues are accustomed to the presence of bacteria and their toxic products, and have become immune in a measure. This fact indicates also that the pathologic manifestations of the gums must have other than a local cause, and we cannot expect to cure all cases of phagedenic pericemtitis by local treatment, either medicinal or prophylactic.

**Arranging Artificial Teeth.** Artificial teeth are usually arranged so that when placed in the mouth they look so uniform and regular that a natural effect is not produced. This should be avoided, because the natural teeth in the mouth are never perfectly uniform. A slight irregularity of two or more teeth adds much to the natural effect that should be produced in artificial dentures, and those who insert them should recognize the importance of the arts as well as the mechanics in prosthetics. Two cases precisely alike cannot be found, and cannot be treated the same with good results in each. We must treat each individual from an individual standpoint. When trying in a plate before vulcanizing, note the effect that a little re-arrangement will produce and in this way good results may be attained.—*Review.*

